# Supplementary material for: Pyridoxamine Alleviates Cardiac Fibrosis and Oxidative Stress in Western Diet-Induced Prediabetic Rats
Source: Int J Mol Sci. 2024 Aug 4;25(15):8508. doi: 10.3390/ijms25158508 (PMC11312841; doi:10.3390/ijms25158508)
Supplement: Supplementary file 1 [file ijms-25-08508-s001.zip › ijms-3062039-supplementary.pdf]

International Journal of *Molecular Sciences*

MDPI

Supplementary material

1. Supplementary tables

Table S1. Supplemental LV echocardiographic and hemodynamic parameters at week 18.

	CD	WD	WD+PM
AWT_d (mm)	2.061 ± 0.119	1.987 ± 0.108	2.217 ± 0.084
AWT_s (mm)	3.453 ± 0.157	3.470 ± 0.187	3.914 ± 0.166
PWT_d (mm)	2.177 ± 0.158	2.109 ± 0.052	2.249 ± 0.052
PWT_s (mm)	3.365 ± 0.204	3.308 ± 0.142	3.565 ± 0.084
IVS_d (mm)	2.061 ± 0.119	1.987 ± 0.108	2.217 ± 0.084
IVS_s (mm)	3.453 ± 0.157	3.470 ± 0.187	3.914 ± 0.166
E/A	1.45 ± 0.18	1.65 ± 0.20	1.30 ± 0.09
E/E′	-26 ± 2	-33 ± 4	-29 ± 3
Longitudinal FS (%)	24 ± 2	18 ± 3	24 ± 2
Radial FS (%)	44 ± 2	45 ± 1	48 ± 2
ID_d (mm)	8.581 ± 0.248	8.895 ± 0.042	8.462 ± 0.186
ID_s (mm)	4.813 ± 0.273	4.861 ± 0.095	4.463 ± 0.242
SV index (μl/cm^2)	0.666 ± 0.028	0.709 ± 0.092	0.634 ± 0.044
Cardiac index (ml/min/cm^2)	0.217 ± 0.015	0.238 ± 0.033	0.218 ± 0.019
EDP (mmHg)	7.1 ± 1.6	8.8 ± 1.0	9.5 ± 1.9
ESP (mmHg)	93 ± 4	98 ± 4	99 ± 2
Tau (s)	0.0116 ± 0.0009	0.0107 ± 0.0015	0.0109 ± 0.0008

Echocardiographic characteristics (upper part of the table) and hemodynamic characteristics (lower part of the table) at the end of the study of CD (n=7), WD (n=4), and WD+PM (n=8). Data represent mean ± SEM. * denotes $p<0.05$ and ** denotes $p<0.01$ vs. CD. AWT_d, anterior wall thickness in diastole. AWT_s, anterior wall thickness in systole. E/A, ratio of peak mitral flow velocity in early versus late filling. EDP, end-diastolic pressure. E/E′, ratio of peak mitral flow velocity versus peak mitral annular velocity. ESP, end-systolic pressure. FS, fractional shortening. ID_d, internal diameter in diastole. ID_s, internal diameter in systole. IVS_d, interventricular septum thickness in diastole. IVS_s, interventricular septum thickness in systole. PWT_d, posterior wall thickness in diastole.

PWT_s, posterior wall thickness in systole. SV, stroke volume. Tau, time constant for isovolumetric relaxation.

Table S2. Primer sequences used for RT-qPCR.

Gene	Forward primer	Reverse primer
Collagen type I A2	GCC AAGAATGCATACAGCCG	GACACCCCTTCTGCGTTGTA
Collagen type III A1	AACTGGAGCACGAGGTCTTG	CGTTCCCCATTATGGCCACT
GPx1	CAATCAGTTCGGACATCAGGAGAA	CTCACCATTCACCTCGCACTT
HPRT	TCCCAGCGTCGTGATTAGTG	GCAAGTCTTTCAGTCCTGTCC
NOX4	TCATGGATCTTTGCCTGGAGGGTT	AGGTCTGTGGGAAATGAGCTTGGA
PGK1	ATGCAAAGACTGGCCAAGCTAC	AGCCACAGCCTCAGCATATTC
RAGE	TGGAAACTGAACACAGGAAGGA	GGAGTGAACCATTGGGGAGG
SOD2	AGCTGCACCACAGCAAGCAC	TCCACCACCCTTAGGGCTCA

Primer sequences are given in their 5′-3′ orientation. GPx1, Glutathione peroxidase 1. HPRT, Hypoxanthine-guanine phosphoribosyl transferase. NOX4, Nicotinamide adenine dinucleotide phosphate oxidase 4. PGK1, Phosphoglycerate kinase 1. RAGE, Receptor for advanced glycation end products. SOD2, Superoxide dismutase 2.

2. Supplementary figures

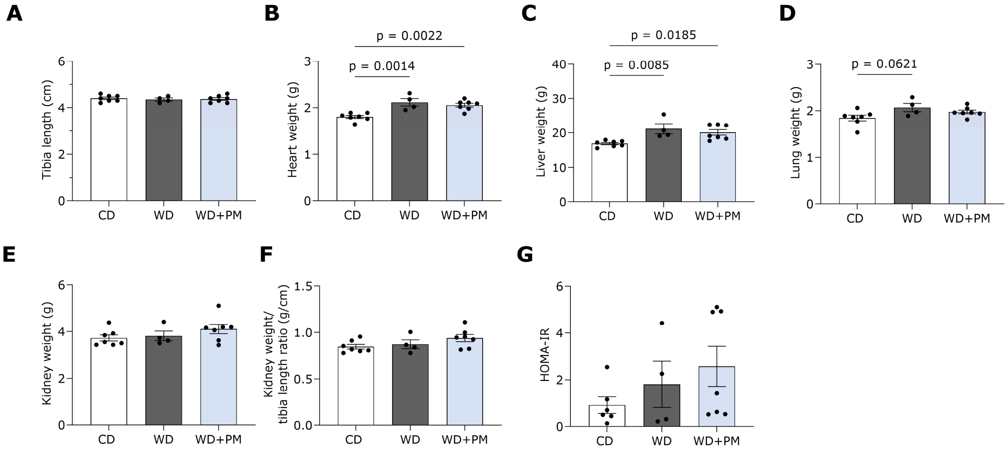

Figure S1. Pyridoxamine does not affect tibia length, kidney weight, and insulin resistance of Western diet-fed rats. **(A)** Tibia length of all groups including CD (n=7), WD (n=4), and WD+PM (n=7). **(B-E)** Absolute values of heart, liver, lung and kidney weight of all groups including CD (n=7), WD (n=4), and WD+PM (n=7). **(F)** Kidney weight to tibia length ratio of all groups including CD (n=7), WD (n=4), and WD+PM (n=7). **(G)** Calculated HOMA-IR index from CD (n=6), WD (n=4), and WD+PM (n=7). Data represent mean ± SEM. HOMA-IR, homeostasis model assessment of insulin resistance.

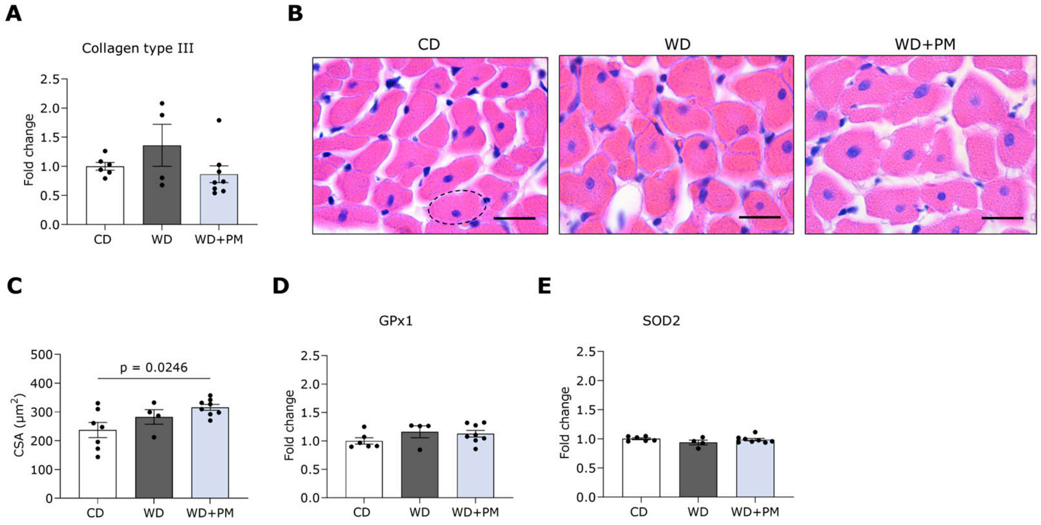

Figure S2. Pyridoxamine does not affect gene expression of collagen type III, cardiomyocyte cross-sectional area and antioxidant enzymes. **(A)** Quantification of gene expression of collagen type III. **(B)** Representative pictures of LV tissue stained with H&E. Scalebar represents 20 μm. The dashed line indicates an elliptical approximation of one cardiomyocyte. **(C)** Quantification of CSA from CD (n=7), WD (n=4) and WD+PM (n=8). Quantification of gene expression of **(D)** GPx1, and **(E)** SOD2 in LV tissue from CD (n=6), WD (n=4), and WD+PM (n=8). Data represent mean ± SEM. CSA, cross-sectional cardiomyocyte area. GPx1, glutathione peroxidase 1. LV, left ventricular. SOD2, superoxide dismutase 2.

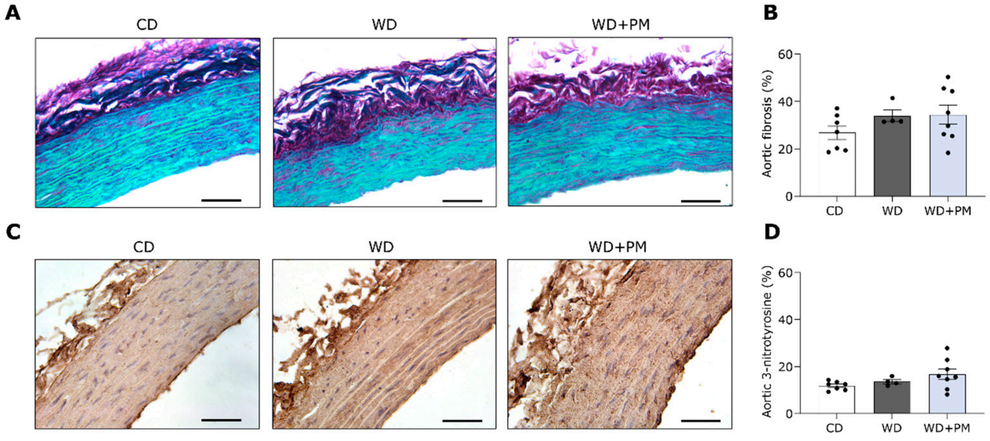

Figure S3. Pyridoxamine does not affect fibrosis and oxidative stress in the aorta of Western diet-fed rats. **(A)** Representative pictures of a Sirius Red/Fast green staining in transverse aortic tissue sections. Red/purple staining indicates collagen deposition or aortic fibrosis. The scalebar represents 50 μm. **(B)** Quantification of collagen deposition in aortic tissue from CD (n=7), WD (n=4) and WD+PM (n=8). **(C)** Representative pictures of 3-nitrotyrosine staining in transverse aortic tissue sections. The scalebar represents 50 μm. **(D)** Quantification of 3-nitrotyrosine in aortic tissue from CD (n=7), WD (n=4) and WD+PM (n=8). Data represent mean ± SEM.
