# Supplementary figures and images for: Pyridoxamine Alleviates Cardiac Fibrosis and Oxidative Stress in Western Diet-Induced Prediabetic Rats
Source: Int J Mol Sci. 2024 Aug 4;25(15):8508. doi: 10.3390/ijms25158508 (PMC11312841; doi:10.3390/ijms25158508)

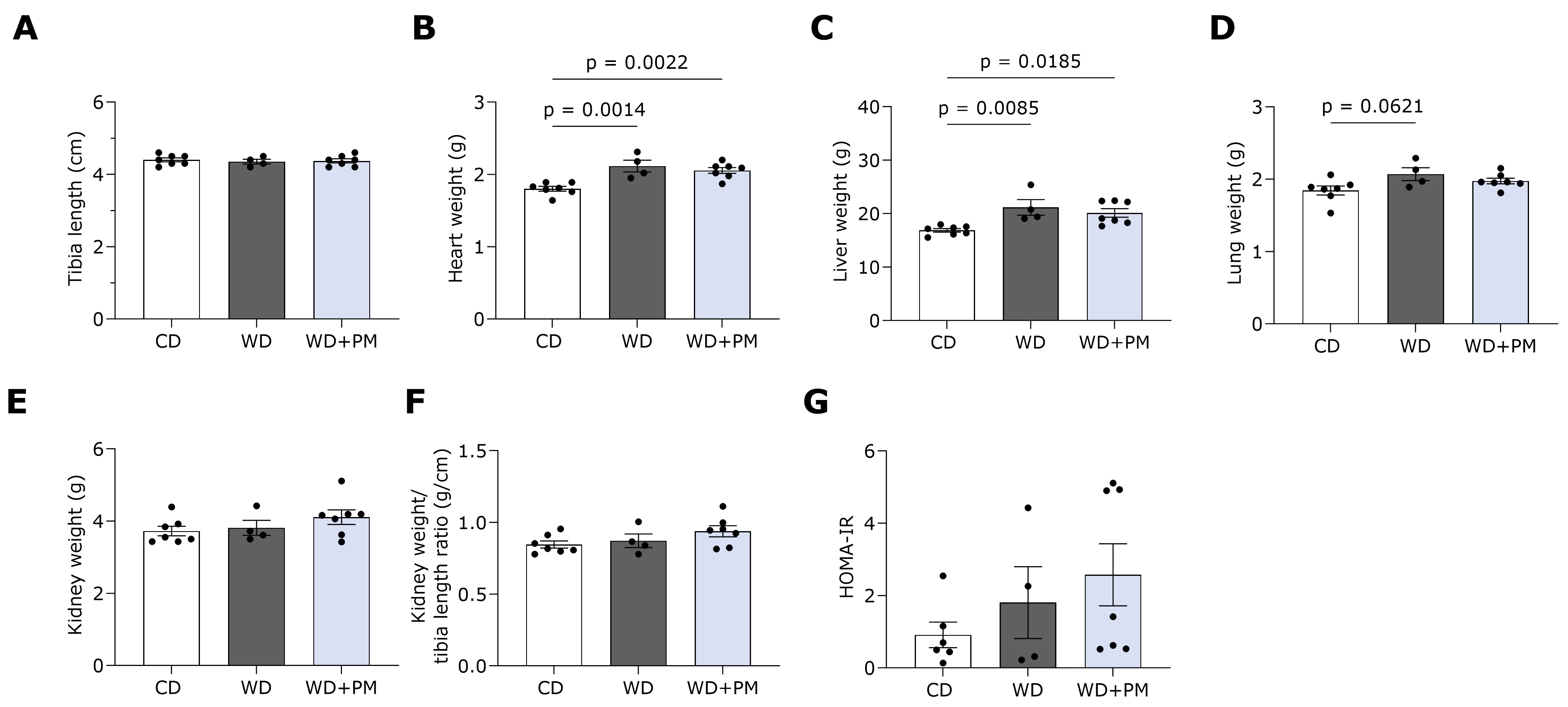

Supplement: Supplementary file 1 [file ijms-25-08508-s001.zip › Figure S1.tif]

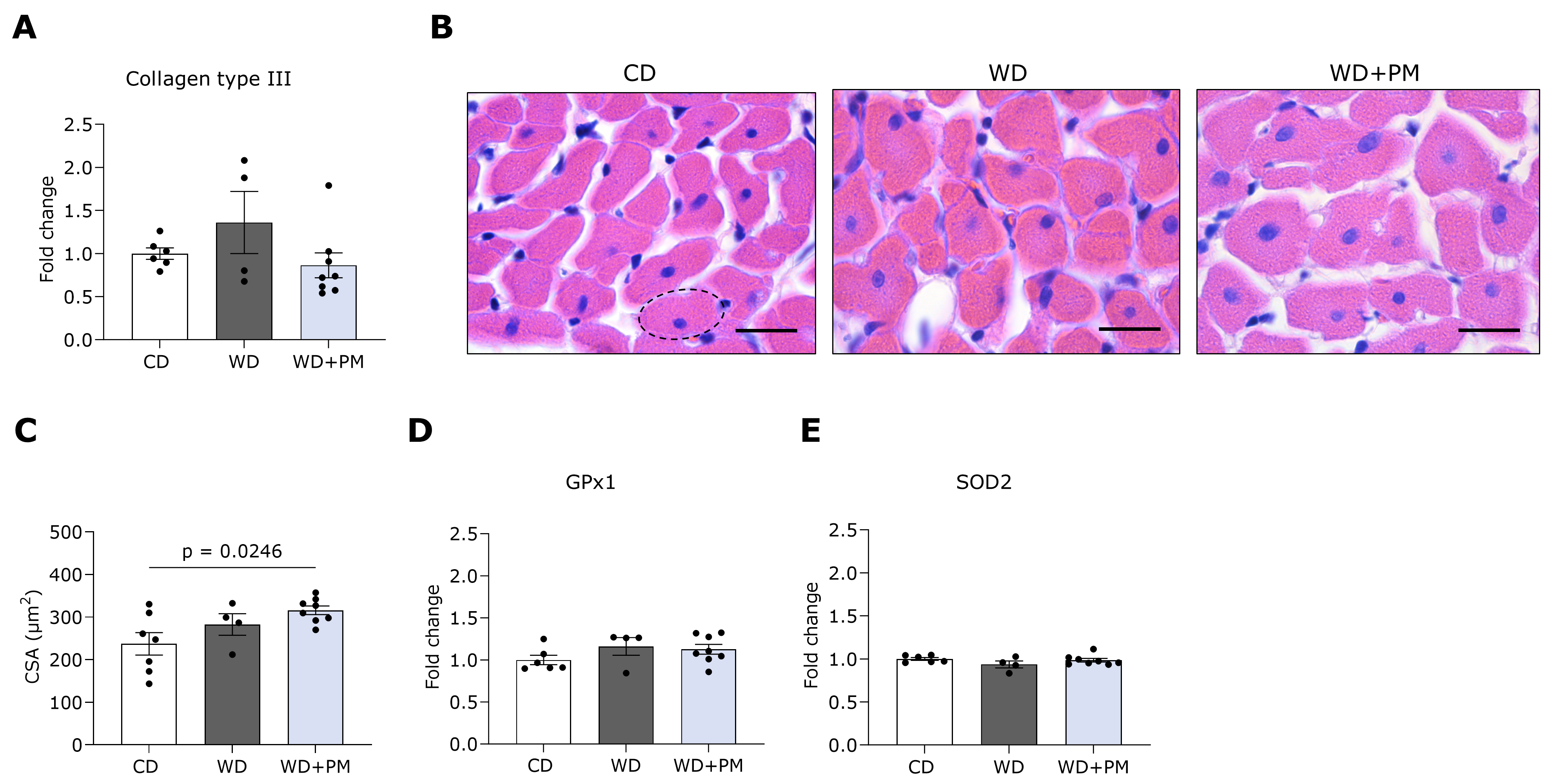

Supplement: Supplementary file 1 [file ijms-25-08508-s001.zip › Figure S2.TIF]

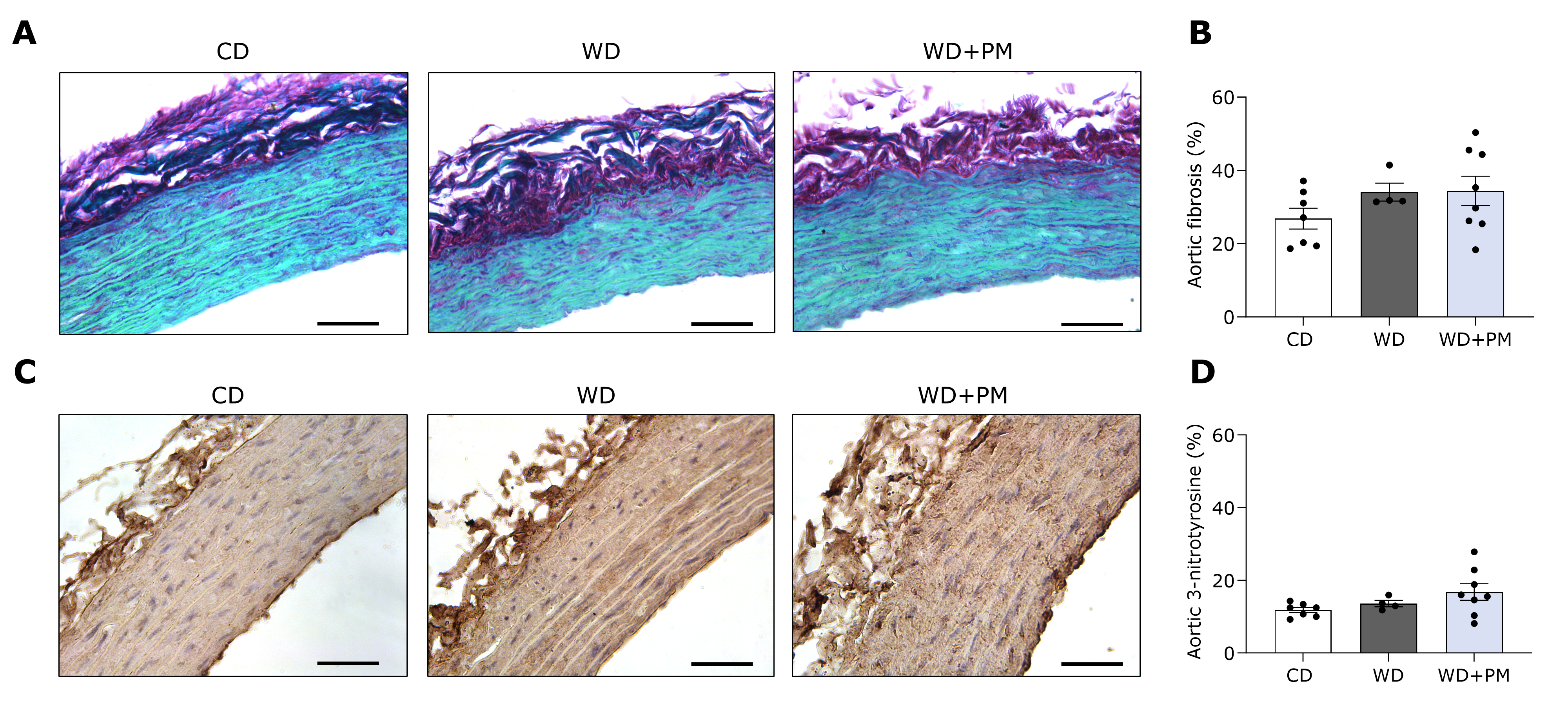

Supplement: Supplementary file 1 [file ijms-25-08508-s001.zip › Figure S3.TIF]
